# Pericoronary adipose tissue imaging: quantitative assessment, artificial intelligence integration, and therapeutic modulation

**DOI:** 10.1093/bjr/tqag040

**Published:** 2026-02-20

**Authors:** Leanne Eveson, Ikboljon Sobirov, Kenneth Chan

**Affiliations:** Division of Cardiovascular Medicine, Radcliffe Department of Medicine, Acute Multidisciplinary Imaging & Interventional Centre, British Heart Foundation (BHF) Centre of Research Excellence, NIHR Oxford Biomedical Research Centre, University of Oxford, Oxford, OX3 9DU, United Kingdom; Division of Cardiovascular Medicine, Radcliffe Department of Medicine, Acute Multidisciplinary Imaging & Interventional Centre, British Heart Foundation (BHF) Centre of Research Excellence, NIHR Oxford Biomedical Research Centre, University of Oxford, Oxford, OX3 9DU, United Kingdom; Division of Cardiovascular Medicine, Radcliffe Department of Medicine, Acute Multidisciplinary Imaging & Interventional Centre, British Heart Foundation (BHF) Centre of Research Excellence, NIHR Oxford Biomedical Research Centre, University of Oxford, Oxford, OX3 9DU, United Kingdom

**Keywords:** pericoronary adipose tissue, inflammation, coronary artery disease, cardiac CT, artificial intelligence

## Abstract

Pericoronary adipose tissue (PCAT) is increasingly recognised as a biosensor of vascular inflammation. The guideline-driven widespread adoption of coronary computed tomography angiography (CCTA) as the first-line investigation for coronary artery disease (CAD) has created opportunities for evaluating the inflammatory burden through quantitative assessment of PCAT. Standardising raw PCAT imaging data for technical, anatomical, and biological variability provides the Fat-Attenuation Index (FAI) Score, which shows promise as a metric of coronary inflammation. Quantification of coronary inflammation has implications for the diagnosis, risk stratification, and monitoring of treatment in atherosclerotic cardiovascular disease. This review examines the anatomical and physiological basis of PCAT, highlighting the importance of standardising PCAT imaging for the implementation as a clinical biomarker, and reviews the role of artificial intelligence (AI) in enhancing precision and scalability. Emerging evidence on the modulation of FAI Score by therapeutic agents, including statins, biologics, and cardiometabolic drugs, and the potential utility of serial imaging in guiding clinical care is also discussed. With ongoing large-scale validation and emerging AI -based approaches, PCAT imaging is poised to complement traditional risk factors and plaque metrics; however, current evidence remains evolving, and the integration of inflammatory risk assessment could be useful to guide emerging anti-inflammatory treatments in personalised cardiovascular medicine.

## Introduction

Coronary artery disease (CAD) remains a leading cause of morbidity and mortality globally.^1^^,^^2^ Coronary computed tomography angiography (CCTA) is a first-line investigation for chest pain symptoms suggestive of CAD.^3–6^ The advent of modern multi-detector CT and photon-counting detector CT with cardiac gating offers high spatial and temporal resolution to visualise coronary artery plaques. This allows the detection of significant luminal obstruction in symptomatic patients, which guides subsequent clinical decisions on coronary interventions to relieve ischaemia. However, a substantial number of acute cardiac events occur from rupture/erosion of smaller non-obstructive plaques.^7^^,^^8^ Although several high-risk plaque features (such as low-attenuation plaque, positive remodelling, spotty calcification, and the napkin ring sign) can help identify vulnerable plaques, these qualitative assessments are limited by modest sensitivity and positive predictive value.^9^ Inflammation is a key pathological process that drives the initiation, progression, and rupture of atherosclerotic plaques.^10–14^ Measuring the activity of coronary inflammation would enable the evaluation of vulnerable vessels and plaques that are at risk of rupturing. This would create an opportunity for early preventive treatment to reduce the risk of acute coronary events.

Translational studies suggest that pericoronary adipose tissue (PCAT) may act as a sensor of vascular inflammation.^10^ PCAT is metabolically active and modulates vascular function through paracrine signalling of adipokines.^15^ In the physiological state, PCAT plays a favourable role in vascular function. In the pathological state, PCAT exhibits pro-inflammatory, pro-oxidative, and pro-atherogenic profiles that promote atherosclerosis.^16^ In turn, inflammatory changes in the vessel drive phenotypic alterations in PCAT, with less well differentiated adipocytes, with increased aqueous: lipid content.^17^ This is detectable non-invasively on routine clinical CCTA with the Fat Attenuation Index (FAI),^17–19^ which shows promise as a quantitative biomarker of coronary inflammation. This review summarises the current understanding of PCAT as a biomarker of coronary inflammation, discusses quantitative CT metrics such as FAI Score, explores the role of AI and radiomic approaches, and outlines emerging therapeutic implications.

## PCAT anatomy and physiology

Adipose tissue is a metabolically active organ with the ability to exert protective and pathogenic effects through the secretion of adipocytokines. PCAT is a biologically distinct sub-depot of epicardial adipose tissue (EAT) with unique paracrine interactions with the vascular wall, implicated in arterial inflammation and atherogenesis.^10^ PCAT is defined as the adipose tissue directly surrounding the vascular wall within a perpendicular distance from the vessel equal to the luminal diameter of the vessel, up to a 2 cm threshold.^10^^,^^20^ For vessels with a luminal diameter greater than 2 cm, perivascular adipose tissue is defined as the adipose tissue within a 2 cm perpendicular distance from the vessel.^10^^,^^11^ In larger coronary arteries, PCAT is often touching the adventitia, whereas in smaller vessels and microvessels, PCAT adipocytes may be incorporated into the vascular wall.

PCAT contains smaller, less differentiated adipocytes with higher physiological macrophage infiltration than subcutaneous fat, and includes a diverse stromal vascular fraction comprising immune, endothelial, neuronal, and mesenchymal cells.^21^ In health, PCAT exerts vasodilatory, anti-inflammatory, and anti-oxidative effects through the release of adipokines, cytokines, and gaseous mediators. It also senses signals from the vessel wall, participating in bidirectional “outside-in” and “inside-out” signalling that regulates vascular tone and homeostasis.^22^^,^^23^

In disease, inflammatory and oxidative mediators from the arterial wall, such as interleukin-6 (IL-6), tumour necrosis factor-α (TNF-α), and lipid peroxidation products, alter PCAT phenotype, promoting lipolysis, impairing adipogenesis, and increasing water content.^10^^,^^24^ These changes produce a gradient of smaller, lipid-depleted adipocytes adjacent to the inflamed vessel, transitioning to larger adipocytes radially outward from the vessel wall. This structural and compositional gradient acts as a “thermometer” of vascular inflammation,^10^ detectable non-invasively on CCTA (Figure 1). Inflamed coronary arteries release lipolytic and inflammatory mediators into the surrounding PCAT, producing smaller, lipid-depleted adipocytes with higher water content near the vessel wall.^17,19^,^25–28^ These compositional changes shift the radiodensity toward the higher (less negative) end of the adipose attenuation range (–190 to –30 Hounsfield Units [HU]) and create a measurable radial gradient. While the biological rationale linking PCAT to vascular inflammation is plausible, CT-derived attenuation metrics represent an indirect surrogate and are influenced by technical, anatomical and acquisition-related factors beyond underlying biology.^29^

**Figure 1.**
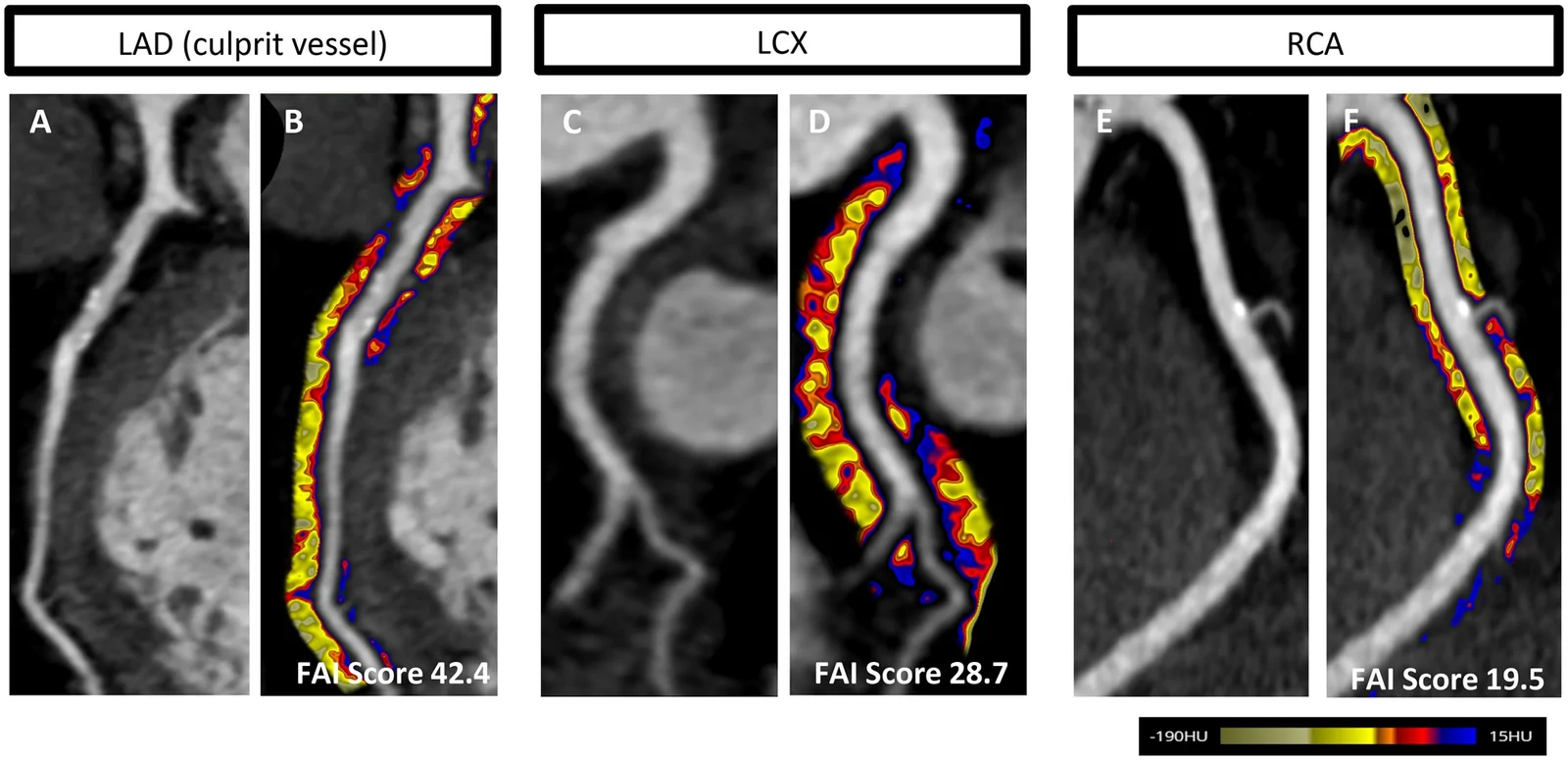
81-year-old male with hypertension presented with acute LAD event 5.1 months after the index CCTA. (A) Non-obstructive mixed plaques in LAD; (B) Perivascular FAI mapping of whole LAD; (C) Left circumflex artery; (D) Perivascular FAI mapping of whole LCX; (E) Calcified plaque in RCA; (D) Perivascular FAI mapping of whole RCA.

### The role of imaging in PCAT assessment

Multiple imaging modalities have been explored for characterising PCAT. CCTA remains the modality of choice, providing high spatial resolution that enable precise quantification of perivascular fat attenuation and texture.^17^ Magnetic resonance imaging (MRI) allows radiation free quantification of epicardial and perivascular fat volume and composition, while simultaneously assessing myocardial structure and function. However, MRI is limited by lower spatial resolution for detailed PCAT analysis. Positron emission tomography (PET), particularly when combined with CT, enables molecular assessment of inflammation through tracers such as ^18F-Fluorodeoxyglucose (FDG) and ^18F-Sodium Flouride (NaF)^30^ which reflect metabolic activity and active microcalcification, respectively. Although PET-CT offers valuable mechanistic insights, its limited spatial resolution, high radiation dose, and restriction to specialist centres limits its wider clinical utility. Overall, CCTA provides the most practical and reproducible approach for routine PCAT quantification, with PET-CT and MRI serving as complementary research tools that deepen biological understanding of vascular inflammation. Table 1 summarises the different imaging modalities for PCAT assessment, highlighting the strengths and weaknesses.

**Table 1.** Imaging modalities for PCAT assessment.

Modality	Strengths	Limitations
CCTA	High spatial and temporal resolution Wide availability 3D volumetric data acquisition	Ionising radiation Iodinated contrast required Attenuation values sensitive to acquisition parameters
MRI	Quantifies epicardial/perivascular fat (thickness, area, volume, proton density fat fraction) No radiation Simultaneous myocardial structure/function assessment Reproducible for large vessel perivascular adipose tissue	Limited spatial resolution for PCAT Limited availability Longer acquisition times High cost
PET-CT	Molecular imaging of inflammation; quantifies SUV and TBR ^18F-NaF detects microcalcification FDG reflects adipose metabolic activity Complements CCTA in research/selected clinical use	Ionising radiation High cost Low spatial resolution (unreliable for PCAT) Limited availability

Abbreviations: 3D = three dimensional, 18F-NaF = 18-Fluorine sodium fluoride, CCTA = coronary computed tomography angiography, FDG = fluorodeoxyglucose, MRI = magnetic resonance imaging, PCAT = pericoronary adipose tissue, PET-CT = Positron emission tomography–computed tomography, SUV = standardised uptake value, TBR = target to background ratio.

CT-based imaging distinguishes normal from inflamed PCAT by analysing changes in fat attenuation and tissue texture that reflect local inflammatory activity in the adjacent vessel wall. Under physiological conditions, PCAT consists predominantly of lipid-rich adipocytes with low attenuation values (approximately −190 to −80 Hounsfield units), corresponding to a high lipid and low aqueous content. During vascular inflammation, cytokine-mediated signalling from the vessel wall alters the metabolic and structural composition of PCAT: adipocytes shrink, lipid content decreases, and there is increased infiltration of water, inflammatory cells, and microvasculature. These changes increase local tissue density, leading to less negative (higher) CT attenuation values.

## Standardising quantitative metrics of PCAT: the fat attenuation index score

PCAT is quantified as an unadjusted mean attenuation of adipose tissue directly adjacent to the coronary arteries, whereas FAI quantifies three-dimensional attenuation gradients considering non-linearly influence of CT values. Initially validated in the proximal right coronary artery (RCA), FAI measurement has subsequently been applied to the proximal left anterior descending (LAD) and left circumflex (LCx) arteries.^19^

In order to further standardise the quantification across different scanner vendors and scanning parameters, the FAI Score corrects FAI values for technical, biological, and anatomical factors, and is interpreted using age and sex specific nomograms.^28^^,^^31^ Harmonisation of these factors helps to ensure the generalisability across different scan parameters, showing promise for yielding a reproducible, platform-independent biomarker of coronary inflammation with prognostic value.

Recent studies have shown that elevated FAI Scores independently predict MACE, even after adjusting for high-risk plaque features, coronary calcium score, and clinical risk factors.^8^^,^^18^^,^^32^ While the FAI score shows potential as an adjunctive biomarker in CAD risk stratification, its role in routine clinical practice remains to be fully established.

## Therapeutic modulation of coronary inflammation and PCAT imaging

In recent years, there has been increasing clinical evidence on a range of treatments that target inflammation to prevent cardiovascular disease.^33^ Imaging, including PCAT, has played a promising role in the identification of individuals with elevated inflammatory risk as well as offering a way to monitor treatment response. A summary of the therapeutic options is detailed in Table 2.

**Table 2.** Impact of medical therapy on coronary inflammation, PCAT attenuation and the fat attenuation index.

Therapy class	Key study	Primary mechanism	Imaging readout	Observed effect on PCAT/FAI	Notes
Emerging or established evidence					
Statins/intensive lipid-lowering	Ridker et al^34–37^	LDL-C lowering plus pleiotropic anti-inflammatory/anti-oxidative effects	CCTA-derived FAI (serial mapping)	↓ FAI (less negative Hounsfield Unit), tracks reduced coronary inflammation; associated with lower MACE risk	FAI changes independent of absolute LDL-C change in longitudinal analyses
Anti-oxLDL biologic (eg, orticumab)	Farina et al^38^	Neutralises oxidised LDL, reduces macrophage activation and vascular inflammation	CCTA-derived FAI; systemic inflammatory markers	↓ FAI with concomitant reduction in systemic inflammation	Early-phase data; supports targeted anti-atherosclerotic strategy
Anti–TNF biologics	Elnabawi^39^	Cytokine blockade (TNF-α), downregulating inflammatory signaling	PET (FDG) coronary inflammation metrics	↓ PET signal of coronary inflammation	Imaging modality differs from FAI; supports biologic anti-inflammatory impact on coronary vasculature
Thoracic radiotherapy (incidental exposure)	Kotanidis et al^40^	Local suppression of perivascular inflammatory activity in irradiated field	CCTA-derived FAI (segmental analysis)	↓ FAI in irradiated coronary segments (sustained)	Breast cancer cohort; highlights PCAT sensitivity to local anti-inflammatory effects
Exploratory evidence					
Colchicine	Event reduction trials (eg, COLCOT^41^, LoDoCo2)^42^; imaging correlates under investigation	Microtubule inhibition; NLRP3 inflammasome modulation	CCTA-derived FAI (ongoing/limited), systemic markers	Putative ↓ PCAT inflammation (imaging confirmation pending)	Proven MACE reduction; mechanistic plausibility for PCAT effects
Canakinumab (anti–IL-1β)	CANTOS^43^; imaging correlates under investigation	IL-1β blockade reducing vascular inflammation	CCTA-derived FAI (limited data), systemic markers	↓ PCAT inflammation (direct FAI evidence pending)	Demonstrated event reduction independent of lipid lowering
SGLT2 inhibitors	Early observational/secondary analyses	Improved cardiometabolic profile; reduced visceral adiposity and systemic inflammation	CCTA-derived FAI (exploratory), body composition metrics	Suggestive ↓ PCAT inflammation	Potential role in secondary prevention; needs dedicated imaging trials
GLP-1 receptor agonists	Early observational/secondary analyses	Weight loss, reduced visceral fat, anti-inflammatory signaling	CCTA-derived FAI (exploratory), body composition metrics	Suggestive ↓ PCAT inflammation	Potential role in secondary prevention; needs dedicated imaging trials

Abbreviations: LDL-C = low density lipoprotein cholesterol, MACE = major adverse cardiovascular events, LDL = low density lipoprotein, TNF = tumour necrosis factor, PET = positron emission tomography, NLRP3 = nucleotide-binding domain, leucine-rich-containing family, pyrin domain-containing-3, IL-1β = interleukin-1 beta, SGLT2 = sodium glucose co-transporter-2, GLP-1 = glucagon like peptide-1.

### Statin and lipid-lowering therapies

Beyond low-density lipoprotein (LDL) cholesterol lowering, statins are known to exert anti-inflammatory effects on the coronary vasculature that leads to improved clinical outcomes.^34–36^^,44^ In a recent prospective CCTA follow-up study, 52 statin-naïve patients with non-obstructive CAD received long-term high-dose statin therapy and underwent serial CCTA at baseline, ∼1 year, and ≥3 years.^37^ There was a significant reduction in FAI Score in the LAD at both follow-ups, a decrease in non-calcified plaque volume and an increase in calcified plaque volume, indicating plaque stabilisation. There was marked improvement in total cholesterol, LDL, and HDL. Higher FAI Score was also correlated with greater non-calcified and lipid-rich plaque volumes. Long-term high-dose statin use in this study reduced coronary inflammation and promoted a shift toward more stable plaque phenotypes.^37^ In this study FAI Score shows promise as a dynamic marker that could be used to monitor the therapeutic impact on coronary inflammation over time.

### Anti-inflammatory therapies

Clinical trial evidence from COLCOT, LoDoCo, and CANTOS has demonstrated the therapeutic potential of anti-inflammatory agents, such as colchicine and canakinumab, in reducing cardiovascular events.^41–43^ These benefits may be mediated, at least in part, by a favourable reduction in coronary inflammation. However, to date, few imaging studies have directly evaluated the effects of such agents on PCAT. Future investigations are warranted to determine whether modulation of vascular inflammation translates into measurable changes in PCAT composition and function.

### Cardiometabolic agents

SGLT2 inhibitors and GLP-1 receptor agonists improve systemic inflammatory profiles and reduce visceral adiposity.^45^^,^^46^ Preliminary data suggest they may attenuate PCAT inflammation, highlighting their potential role in secondary prevention.^47^ More evidence is required in this area to assess the full potential of cardiometabolic agents in modifying vascular inflammation.

### Novel anti-inflammatory agents and biologics

In a pilot randomised, double-blind, placebo-controlled trial in patients with psoriasis, 15 weeks of orticumab, a monoclonal antibody targeting oxidised LDL, significantly reduced coronary inflammation measured by FAI Score on CCTA, particularly among individuals with high baseline inflammation, without safety concerns.^38^ Preliminary findings suggest oxLDL inhibition may lower residual inflammatory cardiovascular risk which can be detected with the FAI Score.^38^

In a prospective observational study of 134 patients with psoriasis, one year of biologic therapy targeting TNF-α, IL-12/23, or IL-17 significantly lowered FAI compared with matched controls, independent of lipid levels or traditional risk factors. The most pronounced effect was observed in the RCA, supporting systemic immune pathway blockade to dampen coronary inflammation.^39^

### Radiotherapy and incidental anti-inflammatory effects

Women with active breast cancer demonstrate elevated predicted cardiac mortality, which could be driven by increased coronary inflammation (elevated FAI Score across all three major coronary arteries) prior to treatment.^40^ Two years after targeted thoracic radiotherapy, both baseline FAI Score and AI-predicted inflammatory risk declined, independent of local cardiac radiation dose.^40^

Across lipid-lowering, immune-modulating, and oxLDL-targeting strategies, reductions in FAI Score measured by CCTA have been observed, often accompanied by favourable changes in plaque composition. Although some of the above studies are preliminary and further evidence is required, these studies collectively show promise in supporting the use of FAI Score as a dynamic biomarker for assessing anti-inflammatory efficacy and guiding precision cardiovascular prevention.

## Role of artificial intelligence (AI) in PCAT imaging

Artificial intelligence (AI) has swiftly advanced medical image analysis, especially through deep learning (DL)-based methods for automatic segmentation. Traditional thresholding and semi-automated methods often encounter problems with limited reproducibility and high operator reliance, making them less suitable for large-scale research. Conversely, convolutional neural networks (CNNs), particularly U-Net^48^ and its three-dimensional variants, have become the main framework for medical image segmentation due to their capacity to capture multiscale spatial features while maintaining fine anatomical details. These models have been effectively adapted for CCTA, allowing for the reliable delineation of vascular and cardiac structures, as well as surrounding adipose tissue, across diverse datasets.^10^^,^^49^^,^^50^ Recent developments, including attention mechanisms, transformer-based modules,^51^ state-space model components,^52^ residual connections,^53^ and enhanced normalisation strategies, have further improved model generalisability and robustness. Moreover, self-supervised pretraining and transfer learning approaches^54^ are increasingly employed to overcome the limited availability of annotated datasets. Collectively, these advances facilitate the creation of consistent, scalable, and fully automated segmentation pipelines that combine PCAT quantification with radiomic or deep feature extraction.

### Deep learning approaches for automatic segmentation

Numerous DL techniques have been developed for analysing coronary arteries and plaques in CCTA scans, offering a potential foundation for segmenting PCAT. Early semi-automated models proved the feasibility of DL-based plaque segmentation but depended heavily on manual input and were limited by small datasets.^55^^,^^56^ More advanced systems, such as hierarchical convolutional long short-term memory (ConvLSTM) network^57^ and 3D U-Net model,^58^ achieved near-expert accuracy in quantifying plaque components, with interclass correlation coefficients above 0.9 for various plaque types. These studies demonstrate the capability of DL architectures to precisely delineate complex coronary structures and tissue compartments in CCTA images.

The same principles apply to PCAT segmentation, where accurately identifying the coronary vessel wall and surrounding adipose tissue is vital for quantifying perivascular attenuation metrics. Several commercial software platforms, such as Autoplaque, CaRi-Heart, CaRi-Plaque, Cleerly, and HeartFlow Plaque Analysis, already incorporate DL-based vascular wall and plaque segmentation for assessing atherosclerosis. CaRi-Heart also integrates fully automated segmentation of PCAT using DL. By adapting these architectures to focus on the perivascular space, DL segmentation offers a reproducible, scalable, automated solution for extracting PCAT, allowing consistent evaluation of perivascular inflammation in large imaging datasets.

### AI-enhanced integration with multi-parametric prognostic models (plaque, coronary inflammation, and clinical risk)

To integrate the information of coronary inflammation in stratifying cardiovascular risk at a patient level, it is important to consider a multitude of prognostic markers of cardiovascular disease, including clinical risk factors as well as plaque burden. AI techniques offer a powerful way to bring these multiple dimensions together into comprehensive multi-parametric models. For instance, DL–based plaque quantification can be combined with FAI measurements to reflect both structural and inflammatory features of coronary disease. Machine learning and survival models can then merge these imaging-derived features with established risk scores (such as Framingham, CAD-RADS or ASCVD) and clinical variables, enhancing patient-level prognosis.

Recent studies have shown that incorporating PCAT signatures alongside plaque burden substantially improves the prediction of major adverse cardiovascular events (MACE) compared to traditional metrics alone.^3^^,^^7^^,^^8^ Radiomic analyses of PCAT add granularity by extracting texture and shape-based descriptors, which may identify subtle tissue remodelling patterns not visible through average attenuation values. Multi-parametric integration represents the natural progression of AI-driven cardiovascular imaging, advancing from single-modality analysis to comprehensive, systems-level disease characterisation. By combining anatomical, functional, and inflammatory data into unified models, AI has the potential to facilitate truly personalised risk assessment and guide targeted therapies.

### External validation in large-cohort studies and population registries and clinical implementation

Translating AI-driven FAI Score analysis into clinical practice necessitates rigorous validation across broad and diverse patient populations. Large-scale registries such as CRISP-CT^32^ and ORFAN^8^ have linked large volumes of CCTA scans with long-term clinical outcomes. These datasets present a valuable opportunity to benchmark AI algorithms in real-world settings, considering variability in imaging protocols, scanners, and patient demographics found in clinical environments. CRISP-CT^32^ was one of the first landmark studies to demonstrate the prognostic importance of FAI Score. By retrospectively analysing patients from Europe and the US, it was shown that FAI Score is an independent predictor of mortality outcomes, beyond plaque burden and conventional risk factors. Importantly, its multi-centre design highlighted consistent associations across healthcare systems using a common analytic framework. More recently, the ORFAN registry^8^ is one of the largest cohorts of patients undergoing CCTA, including over 100,000 CCTA scans (to reach 250,000 scans) linked with long-term clinical outcomes. The size and diversity of ORFAN enable high-dimensional analyses, such as AI-driven segmentation, radiomics, and survival modelling, to be validated across subgroups defined by sex, ethnicity, and comorbidities.

Validation within such registries serves several critical purposes. Firstly, it provides statistical robustness to substantiate the additional prognostic value of FAI Score when incorporated into clinical and imaging-based risk models. Secondly, it facilitates a comprehensive evaluation of the generalisability of algorithms, ensuring that models developed in one population maintain their effectiveness when applied to others. Thirdly, it underpins health-economic modelling, as risk reclassification can be measured across diverse populations to assess the cost-effectiveness of strategies guided by FAI. Finally, registry-based validation establishes an evidentiary framework necessary for incorporation into guidelines and consensus statements, thereby translating the basic sciences to clinical utility.

Prospective validation of PCAT imaging and health-economic modelling for clinical translation is crucial. From a clinical perspective, for the cardiothoracic radiologist who reports CCTA, the emergence of PCAT as a biomarker of CAD should be considered given the downstream treatment implications for patients, especially in the absence of obstructive CAD.

### Fat radiomic profiling with radiotranscriptomic approach

Radiomics is a field that extends beyond simple attenuation by capturing many quantitative features of tissues that capture higher-order textual features invisible to the human eye. Radiomic and AI-driven analyses of PCAT have yielded complementary imaging biomarkers of vascular inflammation. FAI represents a promising dynamic biomarker of vascular inflammation, detecting cytokine-driven changes in PCAT composition.^19^ As discussed, emerging evidence suggests that vascular inflammation as measured by FAI Score can reverse with effective anti-inflammatory therapy. In contrast, the Fat Radiomic Profile (FRP) is an imaging biomarker derived from the surrounding area of the proximal RCA. Unlike FAI, which primarily captures dynamic inflammation-induced changes in PCAT, FRP analyses the PCAT’s radiomic texture and also detects persistent structural remodelling, including fibrosis and microvascular changes, that are indicative of more advanced, irreversible alterations associated with CAD. FRP has been shown to significantly improve the prediction of MACE beyond traditional risk factors and other CCTA features,^59^ and its validity has been demonstrated across multiple cohorts. Its ability to detect long-term adverse remodelling highlights FRP as a robust, non-invasive tool for cardiovascular risk stratification.

Extending this paradigm, radiotranscriptomic signatures such as the C19-RS link CT-derived PCAT features with underlying cytokine activity. The study^60^ introduces a novel AI-assisted radiotranscriptomic biomarker that utilises radiomic analysis of PCAT from the internal mammary artery and descending thoracic aorta, as obtained from routine CCTA, to quantify vascular inflammation driven by cytokines non-invasively. Changes occurring in the PCAT region, as captured by the C19-RS signature, effectively serve as “molecular fingerprints” that characterise and quantify the extent of vascular inflammation. This signature was significantly elevated in COVID-19 patients, particularly those with the B.1.1.7 variant. It demonstrated substantial prognostic value in predicting in-hospital mortality, suggesting that this PCAT-based vascular inflammation could be a key therapeutic target for treatments such as dexamethasone. Together, these biomarkers; FAI, FRP, and radiotranscriptomic signatures, highlight the potential capacity of routine CT angiography to quantify both dynamic and long-term aspects of vascular inflammation, and to connect imaging readouts with underlying molecular pathways, underscoring the power of radiotranscriptomics and AI to translate routine CT imaging into molecular-level biomarkers with broad clinical relevance.

AI-based post processing can improve the consistency of PCAT and FAI measurements; some degree of technical variability related to acquisition, reconstruction, and vendor-specific factors may still remain. Given the proprietary nature of the FAI adjustments, details are not always fully accessible, and concerns might be raised for financial interest. However, as proprietary AI models are regulated as medical devices, locked and subjected to independent regulatory approval processes, confidence in their performance and reliability could be enhanced.

## Technical limitations and future directions

Quantifying PCAT using standard CT methods is limited by several technical and biological factors. Attenuation values are affected by image acquisition settings, such as tube voltage, scanner type, and reconstruction kernel, which differ between centres and over time.^61^ Manual or semi-automated segmentation of PCAT boundaries also introduces operator dependence and inter-observer variation. Moreover, PCAT attenuation only provides a raw measure of inflammation, reflecting overall tissue density. The presence of calcified or fibrotic plaque can alter local attenuation values, making interpretation difficult in atherosclerotic segments. These factors collectively restrict consistency and standardisation across imaging platforms and clinical environments.

AI-based techniques, such as the FAI Score, aim to address some of these challenges by harmonising attenuation measurements across anatomical, biological, and technical factors to create a standardised biomarker of coronary inflammation. Developed across multiple international cohorts and externally validated, the FAI Score shows improved generalisability and reproducibility. However, a key limitation of AI-driven approaches remains explainability:^62^ many DL models function as “black boxes,” providing limited insight into how specific image features influence outcomes. This opaqueness complicates clinical interpretation, model validation, and regulatory approval, particularly when automated outputs are used to guide patient management.

Additionally, the lack of large, well-annotated datasets for PCAT segmentation restricts model training and external validation. Most current studies depend on relatively small, homogeneous cohorts, which limits the ability of models to generalise across different populations, scanners, and acquisition protocols. Overcoming these issues will require the development of standardised datasets, transparent algorithmic frameworks, and collaborative multi-centre validation studies. In summary, although AI-assisted approaches like the FAI Score have greatly improved reproducibility and standardisation in PCAT analysis, further advances in data diversity, interpretability, and regulatory compliance are necessary before widespread clinical adoption can be realised.

Looking ahead, the integration of AI-driven segmentation pipelines with extensive registry data, such as FAI Score and the ORFAN dataset, is expected to facilitate the development of even more robust predictive tools. As federated learning methodologies^63^ advance, it may become feasible to collaboratively train models across various institutions without the necessity of direct data sharing, thereby addressing privacy concerns while maximising sample sizes. Furthermore, prospective registry-based studies, wherein FAI and associated biomarkers actively inform patient management, will be crucial to demonstrate not only predictive accuracy but also tangible clinical benefits. Consequently, large registries serve both as a proving ground and a platform for the validating of AI-enabled multiparametric prognostic modelling (Figure 2).

**Figure 2.**
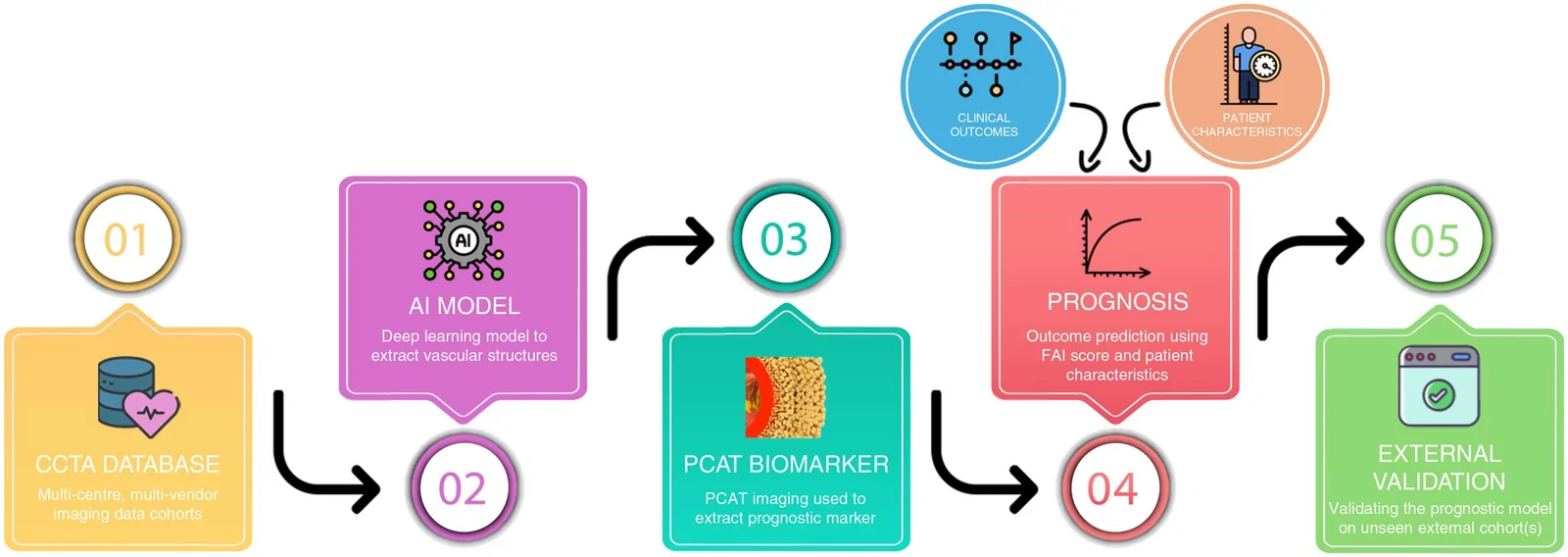
Pathway for AI-enabled multiparametric prognostic modelling for PCAT imaging.

## Conclusion

PCAT imaging represents a biologically plausible and increasingly studied approach to assessing coronary inflammation, although its quantitative standardisation and clinical role continue to evolve. By quantifying inflammatory activity beyond luminal stenosis and plaque morphology, FAI and the complementary radiomic signatures provide novel insights into residual cardiovascular risk that is not captured by traditional risk factors. Serial standardised PCAT imaging offers a potential way to monitor therapeutic response across lipid-lowering, immune-modulating, and cardiometabolic interventions, supporting a precision medicine approach to cardiovascular prevention.

The integration of AI enables automated segmentation, multiparametric risk modelling, and large-scale validation in real-world registries, accelerating translation into clinical practice. As these technologies mature, PCAT imaging is poised to complement established plaque- and risk-based metrics, bridging mechanistic biology with bedside decision-making. Ongoing prospective studies and guideline incorporation will be critical to determine its role in redefining cardiovascular risk assessment and its potential in guiding personalised treatment strategies.

## References

[1] Organisation, W.H. World Health Organization. Cardiovascular diseases (CVDs) [Fact sheet]. Updated 31 July 2025. Accessed September 16, 2025. https://www.who.int/news-room/fact-sheets/detail/cardiovascular-diseases-(cvds)

[2] Zaman S , Wasfy JH, Kapil V, et al The lancet commission on rethinking coronary artery disease: moving from ischaemia to atheroma. Lancet. 2025;405:1264-1312. 10.1016/S0140-6736(25)00055-8 PMC12315672 40179933

[3] The National Institute for Health and Care Excellence (NICE). NICE Guidance for Stable Chest Pain Patients (CG95 & MTG32) to Appropriately Diagnose Patients with Suspected Coronary Artery Disease. 2019.

[4] Knuuti J , Wijns W, Saraste A, et al ESC Scientific Document Group. 2019 ESC guidelines for the diagnosis and management of chronic coronary syndromes: the task force for the diagnosis and management of chronic coronary syndromes of the European society of cardiology (ESC). Eur Heart J. 2020;41:407-477. 10.1093/eurheartj/ehz425 31504439

[5] Fordyce CB , Newby DE, Douglas PS. Diagnostic strategies for the evaluation of chest pain: clinical implications from SCOT-HEART and PROMISE. J Am Coll Cardiol. 2016;67:843-852. 10.1016/j.jacc.2015.11.055 PMC4771619 26892420

[6] Investigators S-H , et al Coronary CT angiography and 5-year risk of myocardial infarction. N Engl J Med. 2018;379:924-933. 10.1056/NEJMoa1805971 30145934

[7] Puchner SB , et al High-risk plaque detected on coronary CT angiography predicts acute coronary syndromes independent of significant stenosis in acute chest pain: results from the ROMICAT-II trial. J Am Coll Cardiol. 2014;64:684-692. 10.1016/j.jacc.2014.05.039 PMC4135448 25125300

[8] Chan K , Wahome E, Tsiachristas A, et al ORFAN Consortium. Inflammatory risk and cardiovascular events in patients without obstructive coronary artery disease: the ORFAN multicentre, longitudinal cohort study. Lancet. 2024;403:2606-2618. 10.1016/S0140-6736(24)00596-8 PMC11664027 38823406

[9] Gallone G , Bellettini M, Gatti M, et al Coronary plaque characteristics associated with major adverse cardiovascular events in atherosclerotic patients and lesions: a systematic review and meta-analysis. JACC Cardiovasc Imaging. 2023;16:1584-1604. 10.1016/j.jcmg.2023.08.006 37804276

[10] Antoniades C , Tousoulis D, Vavlukis M, et al Perivascular adipose tissue as a source of therapeutic targets and clinical biomarkers. Eur Heart J. 2023;44:3827-3844. 10.1093/eurheartj/ehad484 PMC10568001 37599464

[11] Hansson GK. Inflammation, atherosclerosis, and coronary artery disease. N Engl J Med. 2005;352:1685-1695. 10.1056/NEJMra043430 15843671

[12] Libby P. Mechanisms of acute coronary syndromes. N Engl J Med. 2013;369:883-884. 10.1056/NEJMc1307806 23984747

[13] Libby P. Inflammation in atherosclerosis. Nature. 2002;420:868-874. 10.1038/nature01323 12490960

[14] Libby P. The changing landscape of atherosclerosis. Nature. 2021;592:524-533. 10.1038/s41586-021-03392-8 33883728

[15] Szasz T , Bomfim GF, Webb RC. The influence of perivascular adipose tissue on vascular homeostasis. Vasc Health Risk Manag. 2013;9:105-116. 10.2147/VHRM.S33760 PMC3616689 23576873

[16] Lin A , Dey D, Wong DTL, et al Perivascular adipose tissue and coronary atherosclerosis: from biology to imaging phenotyping. Curr Atheroscler Rep. 2019;21:47. 10.1007/s11883-019-0817-3 PMC7172444 31741080

[17] Antonopoulos AS , Sanna F, Sabharwal N, et al Detecting human coronary inflammation by imaging perivascular fat. Sci Transl Med. 2017;9. 10.1126/scitranslmed.aal2658 :28701474

[18] Oikonomou EK , Marwan M, Desai MY, et al Non-invasive detection of coronary inflammation using computed tomography and prediction of residual cardiovascular risk (the CRISP CT study): a post-hoc analysis of prospective outcome data. Lancet. 2018;392:929-939. 10.1016/S0140-6736(18)31114-0 PMC6137540 30170852

[19] Oikonomou EK , Antonopoulos AS, Schottlander D, et al Standardized measurement of coronary inflammation using cardiovascular computed tomography: integration in clinical care as a prognostic medical device. Cardiovasc Res. 2021;117:2677-2690. 10.1093/cvr/cvab286 34450625

[20] Simantiris S , Pappa A, Papastamos C, et al Perivascular fat: a novel risk factor for coronary artery disease. Diagnostics (Basel). 2024;14 10.3390/diagnostics14161830 :PMC11353828 39202318

[21] Kotanidis CP , Antoniades C. Perivascular fat imaging by computed tomography (CT): a virtual guide. Br J Pharmacol. 2021;178:4270-4290. 10.1111/bph.15634 PMC8856184 34296764

[22] Kim HW , Shi H, Winkler MA, et al Perivascular adipose tissue and vascular perturbation/atherosclerosis. Arterioscler Thromb Vasc Biol. 2020;40:2569-2576. 10.1161/ATVBAHA.120.312470 PMC7577939 32878476

[23] Mancio J , Oikonomou EK, Antoniades C. Perivascular adipose tissue and coronary atherosclerosis. Heart. 2018;104:1654-1662. 10.1136/heartjnl-2017-312324 29853488

[24] Mazurek T , Zhang L, Zalewski A, et al Human epicardial adipose tissue is a source of inflammatory mediators. Circulation. 2003;108:2460-2466. 10.1161/01.CIR.0000099542.57313.C5 14581396

[25] Antonopoulos AS , Antoniades C. The role of epicardial adipose tissue in cardiac biology: classic concepts and emerging roles. J Physiol. 2017;595:3907-3917. 10.1113/JP273049 PMC5471417 28191635

[26] Margaritis M , Antonopoulos AS, Digby J, et al Interactions between vascular wall and perivascular adipose tissue reveal novel roles for adiponectin in the regulation of endothelial nitric oxide synthase function in human vessels. Circulation. 2013;127:2209-2221. 10.1161/CIRCULATIONAHA.112.001133 23625959

[27] Antonopoulos AS , Margaritis M, Coutinho P, et al Adiponectin as a link between type 2 diabetes and vascular NADPH oxidase activity in the human arterial wall: the regulatory role of perivascular adipose tissue. Diabetes. 2015;64:2207-2219. 10.2337/db14-1011 25552596

[28] Akoumianakis I , Sanna F, Margaritis M, et al Adipose tissue-derived WNT5A regulates vascular redox signaling in obesity via USP17/RAC1-mediated activation of NADPH oxidases. Sci Transl Med. 2019;11 10.1126/scitranslmed.aav5055 PMC7212031 31534019

[29] Grodecki K , Geers J, Kwiecinski J, et al Phenotyping atherosclerotic plaque and perivascular adipose tissue: signalling pathways and clinical biomarkers in atherosclerosis. Nat Rev Cardiol. 2025;22:443-455. 10.1038/s41569-024-01110-1 PMC12092191 39743563

[30] Kwiecinski J , Dey D, Cadet S, et al Peri-coronary adipose tissue density is associated with (18)F-sodium fluoride coronary uptake in stable patients with high-risk plaques. JACC Cardiovasc Imaging. 2019;12:2000-2010. 10.1016/j.jcmg.2018.11.032 PMC6689460 30772226

[31] Antoniades C , Chan K. Pericoronary adipose tissue imaging and the need for standardized measurement of coronary inflammation: translating PCAT attenuation gradients into FAI score for clinical use. JACC Cardiovasc Imaging. 2025;18:895-898. 10.1016/j.jcmg.2025.06.012 40769621

[32] Oikonomou EK , Desai MY, Marwan M, et al Perivascular fat attenuation index stratifies cardiac risk associated with High-Risk plaques in the CRISP-CT study. J Am Coll Cardiol. 2020;76:755-757. 10.1016/j.jacc.2020.05.078 32762910

[33] Sagris M , Antonopoulos AS, Simantiris S, et al Pericoronary fat attenuation index-a new imaging biomarker and its diagnostic and prognostic utility: a systematic review and meta-analysis. Eur Heart J Cardiovasc Imaging. 2022;23:e526-e536. 10.1093/ehjci/jeac174 PMC9840478 36069510

[34] Ridker PM , Danielson E, Fonseca FAH, et al JUPITER Study Group. Rosuvastatin to prevent vascular events in men and women with elevated C-reactive protein. N Engl J Med. 2008;359:2195-2207. 10.1056/NEJMoa0807646 18997196

[35] Cheng K , Hii R, Lim E, et al Effect of statin therapy on coronary inflammation assessed by pericoronary adipose tissue computed tomography attenuation. Eur Heart J Cardiovasc Imaging. 2025;26:784-793. 10.1093/ehjci/jeaf062 PMC12042745 40037525

[36] Dai X , Yu L, Lu Z, et al Serial change of perivascular fat attenuation index after statin treatment: Insights from a coronary CT angiography follow-up study. Int J Cardiol. 2020;319:144-149. 10.1016/j.ijcard.2020.06.008 32553595

[37] Mátyás BB , Benedek I, Raț N, et al Assessing the impact of long-term high-dose statin treatment on pericoronary inflammation and plaque distribution-a comprehensive coronary CTA follow-up study. Int J Mol Sci. 2024;25:1700. 10.3390/ijms25031700 PMC10855947 38338972

[38] Farina CJ , Davidson MH, Shah PK, et al Inhibition of oxidized low-density lipoprotein with orticumab inhibits coronary inflammation and reduces residual inflammatory risk in psoriasis: a pilot randomized, double-blind placebo-controlled trial. Cardiovasc Res. 2024;120:678-680. 10.1093/cvr/cvae057 PMC11135636 38523341

[39] Elnabawi YA , Oikonomou EK, Dey AK, et al Association of biologic therapy with coronary inflammation in patients with psoriasis as assessed by perivascular fat attenuation index. JAMA Cardiol. 2019;4:885-891. 10.1001/jamacardio.2019.2589 PMC6669789 31365032

[40] Kotanidis CP , Halborg T, Tomlins P, et al Coronary inflammation and cardiovascular risk in breast cancer after radiotherapy. Eur Heart J. 2025;46:2924-2927. 10.1093/eurheartj/ehaf260 PMC12314740 40256882

[41] Tardif J-C , Kouz S, Waters DD, et al Efficacy and safety of low-dose colchicine after myocardial infarction. N Engl J Med. 2019;381:2497-2505. 10.1056/NEJMoa1912388 31733140

[42] Nidorf SM , Fiolet ATL, Mosterd A, et al LoDoCo2 Trial Investigators. Colchicine in patients with chronic coronary disease. N Engl J Med. 2020;383:1838-1847. 10.1056/NEJMoa2021372 32865380

[43] Ridker PM , Everett BM, Thuren T, et al CANTOS Trial Group. Antiinflammatory therapy with canakinumab for atherosclerotic disease. N Engl J Med. 2017;377:1119-1131. 10.1056/NEJMoa1707914 28845751

[44] Ridker PM. The JUPITER trial. Circ: Cardiovasc Qual Outcomes. 2009;2:279-285. 10.1161/CIRCOUTCOMES.109.868299 20031849

[45] Li Y , Yao W, Wang T, et al Association of semaglutide treatment with coronary artery inflammation in type 2 diabetes mellitus patients: a retrospective study based on pericoronary adipose tissue attenuation. Cardiovasc Diabetol. 2024;23:348. 10.1186/s12933-024-02445-2 PMC11439223 39342279

[46] Hara T , Sata M. Pericoronary adipose tissue: potential for pathological diagnosis and therapeutic applications. Cardiovasc Interv Ther. 2025;40:465-473. 10.1007/s12928-025-01126-5 40185991

[47] Morciano C , Gugliandolo S, Capece U, et al SGLT2 inhibition and adipose tissue metabolism: current outlook and perspectives. Cardiovasc Diabetol. 2024;23:449. 10.1186/s12933-024-02539-x PMC11660748 39702365

[48] Ronneberger O , Fischer P, Brox T. U-net: convolutional networks for biomedical image segmentation. In International Conference on Medical Image Computing and Computer-Assisted Intervention, Springer, Cham; 2015:234-241. 10.1007/978-3-319-24574-4_28

[49] Sobirov I , et al Structurally different neural network blocks for the segmentation of atrial and aortic perivascular adipose tissue in multi-centre CT angiography scans. In Annual Conference on Medical Image Understanding and Analysis. Cham: Springer Nature Switzerland.

[50] Ginzburg D , Nowak S, Attenberger U, et al Computer tomography-based assessment of perivascular adipose tissue in patients with abdominal aortic aneurysms. Sci Rep. 2024;14:20512. 10.1038/s41598-024-71283-9 PMC11372190 39227666

[51] Hatamizadeh A , et al UNETR: transformers for 3D medical image segmentation. in Proceedings of the IEEE/CVF Winter Conference on Applications of Computer Vision.

[52] Ruan J , Li J, Xiang S. VM-UNet: Vision Mamba UNet for medical image segmentation. arXiv:2402.02491.

[53] Myronenko A. 3D MRI brain tumor segmentation using autoencoder regularization. In International MICCAI Brainlesion Workshop. Cham: Springer International Publishing.

[54] Ma J , He Y, Li F, et al Segment anything in medical images. Nat Commun. 2024;15:654. 10.1038/s41467-024-44824-z PMC10803759 38253604

[55] Zreik M , van Hamersvelt RW, Wolterink JM, et al A recurrent CNN for automatic detection and classification of coronary artery plaque and stenosis in coronary CT angiography. IEEE Trans Med Imaging. 2019;38:1588-1598. 10.1109/TMI.2018.2883807 30507498

[56] Dey D , Cheng VY, Slomka PJ, et al Automated 3-dimensional quantification of noncalcified and calcified coronary plaque from coronary CT angiography. J Cardiovasc Comput Tomogr. 2009;3:372-382. 10.1016/j.jcct.2009.09.004 20083056

[57] Lin A , Manral N, McElhinney P, et al Deep learning-enabled coronary CT angiography for plaque and stenosis quantification and cardiac risk prediction: an international multicentre study. Lancet Digit Health. 2022;4:e256-e265. 10.1016/S2589-7500(22)00022-X PMC9047317 35337643

[58] Jávorszky N , Homonnay B, Gerstenblith G, et al Deep learning-based atherosclerotic coronary plaque segmentation on coronary CT angiography. Eur Radiol. 2022;32:7217-7226. 10.1007/s00330-022-08801-8 35524783

[59] Oikonomou EK , Williams MC, Kotanidis CP, et al A novel machine learning-derived radiotranscriptomic signature of perivascular fat improves cardiac risk prediction using coronary CT angiography. Eur Heart J. 2019;40:3529-3543. 10.1093/eurheartj/ehz592 PMC6855141 31504423

[60] Kotanidis CP , Xie C, Alexander D, et al ORFAN Investigators. Constructing custom-made radiotranscriptomic signatures of vascular inflammation from routine CT angiograms: a prospective outcomes validation study in COVID-19. Lancet Digit Health. 2022;4:e705-e716. 10.1016/S2589-7500(22)00132-7 PMC9417284 36038496

[61] Jeong D-K , Lee S-S, Kim J-E, et al Effects of energy level, reconstruction kernel, and tube rotation time on hounsfield units of hydroxyapatite in virtual monochromatic images obtained with dual-energy CT. Imaging Sci Dent. 2019;49:273-279. 10.5624/isd.2019.49.4.273 PMC6941831 31915612

[62] Ghassemi M , Oakden-Rayner L, Beam AL. The false hope of current approaches to explainable artificial intelligence in health care. Lancet Digit Health. 2021;3:e745-e750. 10.1016/S2589-7500(21)00208-9 34711379

[63] Zhang C , Xie Y, Bai H, et al A survey on federated learning. Knowl Based Syst. 2021;216:106775. 10.1016/j.knosys.2021.106775

